# Non-Equilibrium Plasma Methods for Tailoring Surface Properties of Polyvinylidene Fluoride: Review and Challenges

**DOI:** 10.3390/polym13234243

**Published:** 2021-12-03

**Authors:** Alenka Vesel, Rok Zaplotnik, Gregor Primc, Miran Mozetič, Tadeja Katan, Rupert Kargl, Tamilselvan Mohan, Karin Stana Kleinschek

**Affiliations:** 1 Department of Surface Engineering, Jožef Stefan Institute, 1000 Ljubljana, Slovenia; rok.zaplotnik@ijs.si (R.Z.); gregor.primc@ijs.si (G.P.); miran.mozetic@ijs.si (M.M.); 2 Institute for Chemistry and Technology of Biobased Systems, Graz University of Technology, 8010 Graz, Austria; tadeja.katan@student-tugraz.at (T.K.); rupert.kargl@tugraz.at (R.K.); tamilselvan.mohan@tugraz.at (T.M.); karin.stanakleinschek@tugraz.at (K.S.K.); 3 Institute of Automation, Faculty of Electrical Engineering and Computer Science, University of Maribor, 2000 Maribor, Slovenia

**Keywords:** polyvinylidene fluoride, gaseous plasma, surface modification, wettability, functionalization, activation

## Abstract

Modification and functionalization of polymer surface properties is desired in numerous applications, and a standard technique is a treatment with non-equilibrium gaseous plasma. Fluorinated polymers exhibit specific properties and are regarded as difficult to functionalize with polar functional groups. Plasma methods for functionalization of polyvinylidene fluoride (PVDF) are reviewed and different mechanisms involved in the surface modification are presented and explained by the interaction of various reactive species and far ultraviolet radiation. Most authors used argon plasma but reported various results. The discrepancy between the reported results is explained by peculiarities of the experimental systems and illustrated by three mechanisms. More versatile reaction mechanisms were reported by authors who used oxygen plasma for surface modification of PVDF, while plasma sustained in other gases was rarely used. The results reported by various authors are analyzed, and correlations are drawn where feasible. The processing parameters reported by different authors were the gas pressure and purity, the discharge configuration and power, while the surface finish was predominantly determined by X-ray photoelectron spectroscopy (XPS) and static water contact angle (WCA). A reasonably good correlation was found between the surface wettability as probed by WCA and the oxygen concentration as probed by XPS, but there is hardly any correlation between the discharge parameters and the wettability.

## 1. Introduction

Polyvinylidene fluoride (PVDF) is a material of choice in any application where relatively light weight and resistance to solvents, acids, and hydrocarbons are demanded, such as in the chemical, semiconductor, medical, and defense industries, as well as in supercapacitors, lithium ion batteries, and Western blots. Like most other fluorinated polymers, PVDF exhibits hydrophobic properties, which are useful in several applications, but rather a drawback in many others, such as membranes for water desalination. The surface properties may be modified by various techniques, including treatment with gaseous plasma [1,2,3,4,5]. The PVDF composition dictates peculiarities in the behavior of this polymer upon exposure to gaseous plasma as compared to other widely used and studied polymers. This paper aims to review the state of the art in tailoring surface properties of PVDF and draw any correlation between the treatment parameters and the surface finish. As reported in the literature, both low-pressure and atmospheric pressure plasmas sustained in different gases have been used for changing the surface morphology and composition of this material.

Non-equilibrium gaseous plasma is a state of gas, rich in charged particles, unstable or metastable gaseous radicals, and radiation. The particles in gaseous plasma assume a Boltzmann distribution over the kinetic energy with the electron temperature ranging from approximately 1 to 10 eV, while the average kinetic energy of neutral molecules, radicals, or ions is much lower, typically below 0.1 eV. The ions’ kinetic energy in gaseous plasma is close to the kinetic energy of neutral molecules, radicals, and atoms because of the efficient energy transfer at elastic collisions. The ions’ kinetic energy upon impinging the surface of any solid object facing plasma is often a few 10 eV and always larger than the average kinetic energy of electrons in a gaseous plasma. The ions’ kinetic energy may be hundreds or even thousands of eV if a polymer object of a reasonable thickness is placed on an electrode powered by a high-frequency voltage source, thus making them the most important plasma particle capable of triggering surface and even subsurface reactions.

Plasma is also a source of radiation in a broad range of wavelengths. The radiation in the far ultraviolet (UV) range (photon energy between approximately 5 and 15 eV) is particularly interesting when plasma is used to tailor the surface properties of polymers, because it can break bonds between the atoms in the solid material. The penetration depth of UV radiation in polymers depends on the type of the polymer and the photon energy and is usually more than a few 10 nm. While the effects of ions and neutral reactive gaseous species are usually limited to a very thin surface film (of the order of 1 nm), unless the polymer is biased by placing on a radiofrequency (RF)-powered electrode, the photons will modify thicker surface films. Many surface reactions are exothermic, so the surface of the polymer is subjected to heating, although the neutral-gas kinetic temperature in gaseous plasma is close to room temperature. The heat is released predominantly on the near-surface region, which stimulates the desorption of light molecules and etching. This effect may suppress functionalization with desired surface functional groups because many groups are thermally unstable.

## 2. Modification of PVDF Surface by Exposure to Argon Plasma

**Figure 1:**
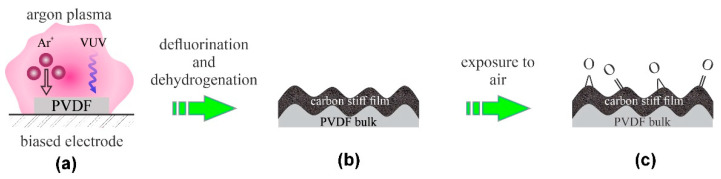
Mechanisms involved in surface modification of PVDF upon exposure to argon plasma sustained by low-pressure capacitively coupled RF discharge: (**a**) polymer is exposed to energetic ions and strong VUV radiation, (**b**) formation of a stiff surface film, and (**c**) oxidation upon exposure to ambient conditions.

Low-pressure plasmas have been used for tailoring surface properties of polymers for decades. One of the first reports on the modification of PVDF by plasma was provided by Duca et al. [6]. They used a low-pressure capacitively coupled RF discharge for sustaining plasma in argon. The plasma reactor was ultra-high vacuum compatible, and it was pumped down to about 4 × 10^−5^ Pa to assure high purity of the atmosphere during plasma treatment. The authors reported the static water contact angle (WCA) for untreated PVDF just above 70°. The WCA dropped to approximately 45° even after a second of plasma treatment and stabilized at approximately 35° after 15 s of plasma treatment. X-ray photoelectron spectroscopy (XPS) showed a gradual decrease in the fluorine concentration. The initial F/C ratio of 0.85 was close enough to the theoretical value (which is 1), and it decreased down to approximately 0.1 for prolonged treatment time at moderate or large powers. The F/C ratio remained approximately 0.5 even after a minute of treatment at the lowest power (4 W) used in their experiments. The oxygen concentration increased after the plasma treatment, which the authors explained by post-treatment oxidation upon exposure to air. No correlation between fluorine depletion and oxygen enrichment was found by Duca et al. [6]. In fact, the highest concentration of oxygen on the Ar-plasma treated PVDF for a minute was found for low discharge powers, i.e., O/C ≈ 0.15. This observation may be a result of the surface heating at elevated powers. The evolution of the surface morphology was monitored by atomic force microscopy (AFM). The roughness increased from 5 to 7 nm after approximately 10 s of plasma treatment at the power of 100 W and remained fairly constant thereafter. More interesting was the observation of the periodical structures on the polymer surface. Duca et al. [6] have not provided any explanation for the appearance of the periodical structures, but a couple of decades later, Bruce et al. [7] explained such structures by the formation of a stiff plasma-modified film on the soft unmodified bulk. Furthermore, Vegh et al. found out that the Ar^+^ ions cause dehydrogenation and crosslinking of the modified layer because of the preferential sputtering of H atoms relative to C atoms [8]. Recently, Lojen et al. reported the influence of the vacuum ultraviolet (VUV) radiation on the surface kinetics of fluorinated polymers upon plasma treatment [9]. The recent achievements in the field of plasma–polymer interaction [8,9] enable a detailed explanation of results reported by Duca et al. in their pioneer paper [6]. Upon exposure of PVDF to plasma sustained in high-purity argon by capacitively coupled RF discharge, the PVDF material is subjected to both energetic Ar^+^ ions and VUV radiation. Both ions and radiation cause depletion of the surface film from fluorine and hydrogen, thus causing a thin surface film of periodical morphology. The surface film is rich in carbon after the plasma treatment. The carbon-rich surface film is then partially oxidized upon exposure to ambient air prior to XPS characterization. The effect is illustrated in Figure 1.

A few years after the pioneering work by Duca et al. [6], Park et al. probed the surface modification of PVDF by argon plasma [10]. Unlike Duca, Park et al. [10] used an electrodeless configuration of the RF discharge, so the PVDF samples were not biased but left at the floating potential during the plasma treatment. The Ar^+^ ion kinetic energy was therefore much smaller. Other parameters were similar: Park et al. [10] also selected the pressure of 13.3 Pa, the discharge power was varied between 25 and 100 W, and the treatment time between 10 and 180 s. The PVDF samples were placed away from the main discharge zone, so both the plasma density and VUV radiation were smaller than in the case of Duca’s configuration [6]. Not surprisingly, the observed surface finish was much different. First, the periodical morphology was not observed. In fact, the roughness as determined by AFM even decreased after the argon plasma treatment performed by Park et al. [10]. The WCA decreased marginally after the treatment at low powers and stabilized at approximately 80° when the samples were treated at 25 W. Most experiments by Park et al. [10] were performed at the power of 100 W. At this power, the WCA decreased to 82°, 55° and 56° after treating the sample for 10, 60 and 180 s, respectively. The remote argon plasma was therefore not very efficient for hydrophilization of PVDF. The F/C ratio decreased from the original 0.93 to 0.49, 0.51 and 0.42 after treating the sample for 10, 60 and 180 s, respectively. The O/C ratio, however, increased significantly. The ratio was immeasurably low for an untreated sample but increased to 0.18, 0.22 and 0.23 after treating the sample for 10, 60 and 180 s, respectively. The huge discrepancy between the results reported by Duca et al. and Park et al. [10] can be explained by differences in plasma parameters. The synergetic effects between energetic Ar^+^ ions and VUV radiation used by Duca et al. [6] caused extensive surface modifications, in particular, the almost complete defluorination of the surface layer. The surface became rich in carbon which could not be oxidized well upon exposure to ambient air. On the other hand, weak argon plasma used by Park et al. [10] was not as an extensive source of radiation as the powerful plasma of Duca [6]. Furthermore, the kinetic energy of Ar^+^ ions impinging PVDF at a floating potential is marginal to the energy at biased surfaces. As a result, some fluorine persisted in the surface film, so the wettability remained rather poor even after prolonged treatment with argon plasma. In contrast, the incomplete dehydrogenation of the surface film favored oxidation upon exposure to air. As recently predicted by theory [11] and confirmed by experiments [12], the C–H bond is replaced by C–OH even after receiving a very small fluence of O atoms. The discrepancy between the wettability and the oxygen concentration between the results reported by Duca et al. [6] and Park et al. [10] reveals a non-trivial relationship between these two properties of argon plasma-modified fluorine-containing polymers. Park et al. [10] also probed oxygen plasma treatment in the same experimental system and found practically no improvement in wettability. The paradox will be explained later in this manuscript.

**Figure 2:**
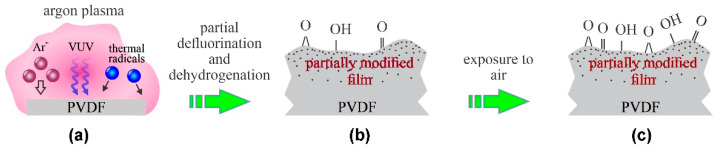
Mechanisms involved in surface modification of PVDF upon exposure to diffusing argon plasma with samples kept at the floating potential: (**a**) polymer is exposed to slow ions, weak VUV radiation and radicals or residual atmosphere, (**b**) formation of a gradient surface film and radical-dependent surface oxidation, and (**c**) full surface oxidation upon exposure to ambient conditions.

The diffusing argon plasma was also used for the treatment of PVDF membranes prepared by electrospinning [13]. The membranes consisted of randomly distributed fibers of a typical diameter of a little more than 100 nm. Argon plasma was sustained by a remote microwave (MW) discharge, so the samples were kept at the floating potential. Rough pumping of the plasma system was performed; therefore, the residual atmosphere rich in water vapor persisted throughout the argon-plasma treatment. The water vapor was effectively dissociated upon plasma conditions, and the resulting OH and O radicals interacted with the PVDF fibers to cause replacement of the surface C–H bonds with the C–OH, thus forming polar groups as confirmed by Fourier-transform infrared spectroscopy (FTIR). A prolonged treatment time of about 5 min was reported by Yalcinkaya et al. [13], and the resultant surface functional groups caused hydrophilization so that a water droplet penetrated into the membrane before the WCA could be measured. The surface finish was, therefore, similar to that illustrated in Figure 2.

**Figure 3:**
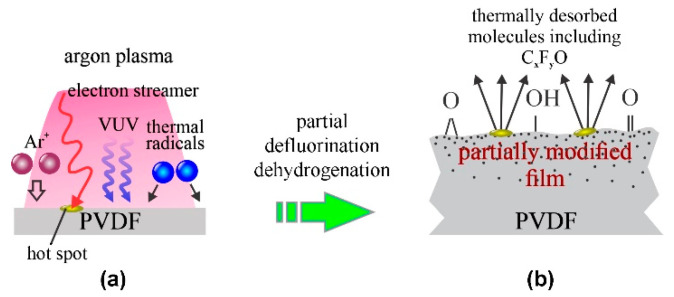
Mechanisms involved in surface modification of PVDF upon exposure to atmospheric-pressure argon jet: (**a**) polymer is exposed to slow ions, weak VUV radiation and radicals or residual atmosphere, temporally and spatially limited hot spots appear at the surface where electron streamers impinge, and (**b**) formation of a gradient surface film, radical-dependent surface oxidation and etching at hot spots.

In addition to low-pressure plasmas, atmospheric pressure argon plasmas were used for treating PVDF materials [14,15]. An extensive paper was published by Akashi and Kuroda [14]. They used a standard argon atmospheric pressure plasma jet (APPJ) sustained by a dielectric barrier discharge (DBD). Such a configuration has been used by numerous authors for the treatment of a variety of polymers. The physical dimensions of APPJ enable only the modification on a surface spot of a typical diameter of a few mm [16]. Despite a high argon purity, plasma sustained in such configuration is a rich source of oxidizing radicals [17], so the surface finish may depend on the concentration of the radicals (in particular OH and O in the plasma plume). Akashi and Kuroda [14] reported a complex activation mechanism starting with dehydroflourination and/or defluorination, followed by etching and functionalization with oxygen-containing functional groups. As a result of these reactions, the surface morphology changed upon the plasma treatment. The WCA decreased monotonously with increasing treatment time. At the discharge voltage of 3 kV, the WCA decreased from the original 120° to approximately 100°, but at 5 kV to approximately 85° after several minutes of plasma treatment. The F/C ratio as determined by XPS followed the WCA trend, and the minimal achievable F/C was approximately 0.3. Interestingly enough, the O/C ratio quickly increased to approximately 0.2 and remained unchanged thereafter despite the increasing F/C ratio. The evolution of the surface composition as probed by XPS for the case of APPJ [14] is therefore similar to that observed for diffusing low-pressure argon plasma [10] and different from the results reported by Duca et al. [6]. The APPJ lacks energetic Ar^+^ ions impinging the polymer surface, so the surface kinetics upon treatment of the PVDF with atmospheric-pressure Ar-plasma are similar to those illustrated in Figure 3. 

The key difference between low-pressure and atmospheric plasma is that the latter (sustained by DBD) consists of numerous electron streamers which stochastically impinge the surface and thus form localized hot spots [18]. The high temperature at these spots causes significant etching. The role of gaseous impurities (in particular water vapor) on the surface functionalization of PVDF upon treatment with the argon APPJ is yet to be elaborated, but the results reported for other polymers reveal the VUV irradiation is the key mechanism enabling the activation of polymers using atmospheric-pressure Ar jets [19]. Akashi and Kuroda published another paper revealing similar results, except that the initial F/C ratio was much different [15].

All results reported by various authors who tackled surface modification of PVDF by treatment with argon plasma are summarized in Table 1.

## 3. Modification of PVDF Surface by Exposure to Oxygen Plasma

A natural choice for the functionalization of polymers with oxygen-containing functional groups is an application of oxygen plasma. This technique was applied by several authors. An early work was published by Vandencasteele et al. [20]. They used diffusing oxygen plasma sustained in a Pyrex bell jar by an RF discharge. The reactor was pumped thoroughly by a turbomolecular pump, so the residual atmosphere was negligible. High purity gas was then introduced into the reactor during continuous pumping, so the reactive gas pressure was 7 Pa during exposure of the PVDF samples to plasma. The samples were placed away from the electrodes, so they were kept at the floating potential. The WCA decreased from the initial 70° down to approximately 10° after treating for 10 min at the discharge power of 20 W. The F/C ratio followed the evolution of the WCA and approached approximately 0.2, whereas the O/C approached 0.4. The initial F/C ratio as probed by XPS was 0.6, so far from the theoretical value, which is 1. The authors explained the discrepancy as an artefact of the XPS measurements or contaminants that could not be removed by classical chemical cleaning. The authors [20] proposed the removal of HF molecules from the PVDF surface during the treatment with oxygen plasma and the replacement of the C-F bond with an oxygen functional group. The model proposed in [20] was based on a detailed study of the evolution of the C1s XPS peak, which clearly showed the gradual disappearance of the CF_2_ functional group.

A Pyrex jar plasma reactor was also used by Kim et al. [21] to treat PVDF samples with oxygen plasma. A capacitively coupled RF discharge operating at the power of 10 W sustained plasma, but the samples were kept at a floating potential. The treatment time was between 10 and 180 s. The experimental configuration was almost identical to that in [20]. The samples (PVDF membranes) were carefully cleaned with ethanol in a sonicator, and the initial WCA (after the cleaning and before plasma treatment) was 120°. The WCA dropped to approximately 80° after 10 s of plasma treatment and stabilized at approximately 70° for prolonged treatment. The XPS characterization revealed an F/C ratio of 0.8 for untreated samples. The oxygen plasma treatment caused a weak defluorination because the F/C ratio dropped only to 0.76. Simultaneously, the O/C ratio of 0.07 was observed. Such a rather poor functionalization was explained by etching upon treatment of PVDF with oxygen plasma. Kim et al. [21] suggested that oxygen plasma etching could chop off the polymer chain and thus degrade the polymer material into the oligomers to form weak boundary layers on the surface. They provided high-resolution C1s and O1s peaks to support the conclusion about the preferential etching of the C–H_2_ components on the PVDF surface. Both hydroxyl and carbonyl groups were detected on the surface of samples treated with oxygen plasma. 

Park et al. [10] also probed oxygen plasma treatment of PVDF foils. As explained in Section 2, the foils were placed away from the main discharge zone, so they were subjected to a weak diffusing plasma. Such conditions enabled a marginal decrease in the static WCA after a few 10 s intervals of oxygen plasma treatment. Further treatment had no effect on the WCA, at least not in the range of the probed powers and treatment times, i.e., 25–70 W and 30–180 s. The authors found only weak defluorination of the foils after oxygen plasma treatment and concluded that argon plasma performs better as long as the defluorination or substitution of fluorine with oxygen is the merit. The paradox was explained almost two decades later by Primc [22], who stressed that the oxygen–plasma treatment causes the etching of fluorine-containing polymers rather than functionalization with oxygen-functional groups.

Correia et al. [23] also used capacitively coupled RF plasma, except that the plasma was sustained in a stainless steel reactor. The samples of electrospun PVDF fibers were placed onto the grounded housing of the plasma reactor. The discharge power was varied between 120 and 480 W. The optimal conditions for improved hydrophilization were found at the treatment time of 2 min, and the discharge power of 360 W. XPS characterization revealed a F/C ratio of 0.94 for untreated samples, 0.67 for samples treated at 240 W and 0.77 for samples treated at 360 or 480 W. A simple model of plasma–surface interaction was proposed. Unlike many other authors, Correia et al. [23] proposed the substitution of a fluorine atom bonded to carbon by the COOH group and spontaneous decay of this group to form either a carbonyl or hydroxyl group. They also measured water contact angles and provided a procedure for obtaining hydrophilic membranes: at the treatment time of 2 min, the WCA did not change much from the original 135° until the discharge power assumed 360 W. Thereafter, the membranes became so hydrophilic that the water droplet absorbed inside the membrane, so it was not possible to measure the static WCA. Keeping the discharge power fixed at 360 W, the WCA remained fairly unchanged up to the treatment time of approximately 80 s, and the water was absorbed after longer treatment times. The transformation from the hydrophobic to hydrophilic surface properties was therefore abrupt—either the WCA was around 135° or the water droplet was absorbed in a time short enough to prevent measuring the contact angle. The soaking of a water droplet into an electrospun PVDF membrane was also reported by Rodrigues after 5 min of treatment with oxygen plasma at a pressure of 20 Pa [24]. Unfortunately, no details about the discharge configuration were provided.

Jeong et al. [25] also used capacitively coupled RF plasma to sustain oxygen plasma for modification of surface properties of PVDF membranes. The treatment time was between 20 and 120 min. Plasma was sustained in a metallic chamber, and the samples were placed on the grounded electrode. The grounded electrode was connected to the grounded housing, so the asymmetric configuration of the RF discharge assured moderate kinetic energy of positive ions impinging the polymer surface. The oxygen pressure was fixed at 2.7 Pa. The discharge power was 62 W. The surface composition was probed by energy-dispersive X-ray spectroscopy (EDX). This technique is not very surface sensitive, so the reported composition cannot be compared with results obtained by XPS. For the sake of paper completeness, we included the results of Jeong et al. [25] in Table 1. Interesting enough, the pristine samples exhibited a F/C ratio as low as 0.5, but half an hour of plasma treatment caused an increase of the F/C to the theoretical value of PVDF, i.e., F/C = 1. Two hours of treatment caused the F/C ratio to = 0.6.

All results reported by various authors who tackled surface modification of PVDF by treatment with oxygen plasma are summarized in Table 2.

The reactions reported by different authors who have tackled treatment of polyvinylidene fluoride with oxygen plasma are summarized as follows:

- Weak or moderate depletion of fluorine (all authors);
- Moderate or weak enrichment of the surface with oxygen (all authors);
- Weak or moderate hydrophilicity (all authors);
- Desorption of HF molecules [20];
- Polymer chain chopping [21];
- Formation of carboxyl group [23];
- Formation of carbonyl, hydroxyl or epoxy functional group [20,21,23];
- Etching (at least implicitly expressed by all authors).

The mechanisms suggested by different authors are difficult to compare as plasma parameters (the density of charged and neutral reactive particles, the intensity of VUV radiation) were not reported. Still, oxygen plasma is always an extensive source of neutral oxygen atoms in the ground state [26], which readily interact with polymer surfaces irrespective of other reactants. A high dissociation fraction of oxygen molecules persists even in late afterglows, where other reactants are absent. Oxygen plasma is also a source of VUV radiation at about 130.4 nm arising from the resonant transition of O atoms [27], although the radiation is not as extensive as that from argon plasma at identical conditions [28]. The two necessary requirements for surface activation are therefore satisfied when using oxygen plasma: VUV radiation for breaking C–F bonds or the polymer chain and O atoms for the occupation of the dangling bonds. The rather poor surface activation reported by the authors who compared results obtained by argon and oxygen plasmas at identical conditions may be explained by the etching of already functionalized surfaces.

According to theoretical predictions [11], the substitution of hydrogen with the hydroxyl group is highly probable and exothermic. The initial stage in oxygen-plasma treatment of the PVDF materials should be the partial substitution of the surface C–H bonds with the C–OH functional groups. Such substitution will not influence the F/C ratio. All authors, however, reported fluorine depletion from the surface film as probed by XPS even at the shortest treatment times and/or smallest discharge power. The initial stage of the PVDF surface modification by oxygen-plasma treatment, therefore, remains to be studied.

The depletion of fluorine from the surface of PVDF, as reported by all authors, should be the consequence of the VUV radiation. Such a depletion was also reported by authors who kept the polymer samples at a floating potential where the kinetic effects caused by O_2_^+^ (or O^+^) ions are negligible. The F atom may interact with neighboring hydrogen from the C–H bond to form an HF molecule which is desorbed from the surface in vacuum conditions. This mechanism was suggested in [20]. Oxygen atoms probably occupy the dangling bonds. Further treatment will cause the formation of various functionalities rich in oxygen and the desorption of various low-mass molecules containing fluorine, oxygen, carbon and/or hydrogen. This effect causes etching. The exact PVDF etching mechanisms relevant for oxygen-plasma treatment of this polymer are still unknown and yet to be discovered.

## 4. Modification of PVDF Surface by Plasma Sustained in Other Gases or Gas Mixtures

An alternative to oxygen plasma is an application of low-pressure non-equilibrium plasma sustained in a mixture of argon and reactive gases such as hydrogen, nitrogen, oxygen, etc. Such plasmas are extensive sources of excited species capable of chemical interaction with polymer surfaces [29,30]. A gas mixture of argon and carbon dioxide was used by Gopakumar et al. [31] for the treatment of PVDF electrospun membranes. The plasma reactor was first evacuated with a rotary vacuum pump with the nominal pumping speed of 10 m^3^/h and the authors managed to obtain the base pressure of 0.13 Pa. High-purity Ar and CO_2_ were leaked into the vacuum system, so the operating pressure was 60 Pa. The CO_2_ partial pressure was 13 Pa. Plasma was sustained with an MW generator with a nominal power of 1000 W. The samples were placed into the quartz tube a few cm away from the MW resonator, so at a similar position as adopted by Yalcinkaya et al. [13], who used pure Ar plasma. The treatment time was 5 min. FTIR revealed the formation of C=O and C–O functional groups during the plasma treatment. The functionalization might be a consequence of the effects of argon-born species, similar to the results of Yalcinkaya et al. [13], who used an identical discharge configuration but only pure argon. The significant partial pressure of CO_2_, however, caused O-atom rich plasma due to the partial dissociation of the CO_2_ molecules [32], and the oxygen atoms might have bonded to the modified surface layer. Only a moderate decrease in the hydrophobicity was observed because the WCA dropped from the initial 140° to approximately 100°. Excellent filtering capability was reported.

Recently, Kormunda et al. [33] probed the hydrophilization of electrospun PVDF fibers using gaseous plasma sustained in the air at a relative humidity of 32% at atmospheric pressure and room temperature. The standard DBD discharge was powered by a 3 kHz, 120 W sinusoidal source operating at the peak-to-peak voltage of 20 kV. The plasma was in filamentary mode, the same as the Ar plasma jet used by Akashi and Kuroda [14]. The XPS survey spectra indicated an interesting composition of as-prepared PVDF fibers: 57 at.% C, 25 at.% F, 11 at.% O and the rest were elements such as phosphorus, potassium and sodium. These elements were probably incorporated into the polymer fibers upon preparation using the wire-spinning technique. The plasma treatment times were between 0.5 and 60 s. Na and K were gradually disappearing from the surface film probed by XPS during the plasma treatment, but P was more persistent. The C concentration was decreasing with increasing plasma treatment time down to about 48 at.%. Interestingly enough, the O concentration in the surface film was not affected by the plasma treatment, and no nitrogen was observed despite using air plasma. On the other hand, the F concentration was found to increase with the increasing plasma treatment time. Even more interesting was the behavior of the surface wettability: the initial WCA as determined after the synthesis and before the plasma treatment was as low as 69°. A few seconds of plasma treatment caused an increase of the WCA to the values typical for highly porous non-activated PVDF, i.e., about 130°. Further treatment caused an instant loss of the hydrophobicity, the same effect as previously reported by Correia et al. [23] and Rodrigues et al. [24]. The latter authors used low-pressure oxygen plasma. The results reported by Kormunda et al. [33] might be explained by surface etching upon plasma treatment. The filamentary plasma causes localized hot spots on the polymer sample, so the surface temperature is increased at the position where a streamer touches the polymer surface (Figure 3). Any polar functional group that might have formed upon the interaction of plasma radicals such as carbon bonded to O, N and OH, desorbs from the surface, leaving the most resistant component (i.e., C–F bonds) on the surface. This effect may explain the rather unexpected increase in the F concentration with increasing plasma treatment time. The detailed analyses of high-resolution C1s and O1s spectra confirmed this speculation: a significant amount of oxygen on untreated samples was found bonded in the form of metal oxides, so the concentration of oxygen bonded to carbon increased with increasing plasma treatment. The C–OH and C=O groups contribute to increased hydrophilicity, so when the impurities are removed from the fibre surfaces, the material loses its hydrophobicity and soaks up water droplets.

Vandencasteele et al. also probed modification of PVDF surfaces using nitrogen plasma [20]. Their experimental system has already been described in Section 3. They found a gradual activation of the surface with increasing treatment time, but the final WCA did not drop below 18°, at least not for treatment times up to 10 min at the discharge power of 20 W. Unfortunately, they did not report the surface composition versus the treatment time. 

Apart from improved wettability, which is obtained by grafting oxygen functional groups onto a polymer surface, other surface functionalities may be required. In their classical paper, Müller and Oehr [34] studied the functionalization of PVDF membranes with amino groups. They used a pulsed RF discharge operating at a pressure of 20 Pa using an electrodeless coupling. They selected allylamine, diaminocyclohexane, ammonia, and a mixture of hydrogen and nitrogen to sustain the gaseous plasma in a quartz tube. They reported little or no functionalization with the primary amino groups when using ammonia or a mixture of hydrogen and nitrogen but satisfactory results when the more complex gases were used. Best results were observed at the lowest power (1 W), which was explained by grafting rather than substitution of surface functional groups. The organic precursors were partially dissociated upon plasma conditions, and the radicals stuck onto the surface to form very thin films rich in amino groups.

All results reported by various authors who tackled surface modification of PVDF by treatment with a plasma sustained in gases other than argon or oxygen are summarized in Table 3.

## 5. Correlations between Processing Parameters and the Surface Finish

**Figure 4:**
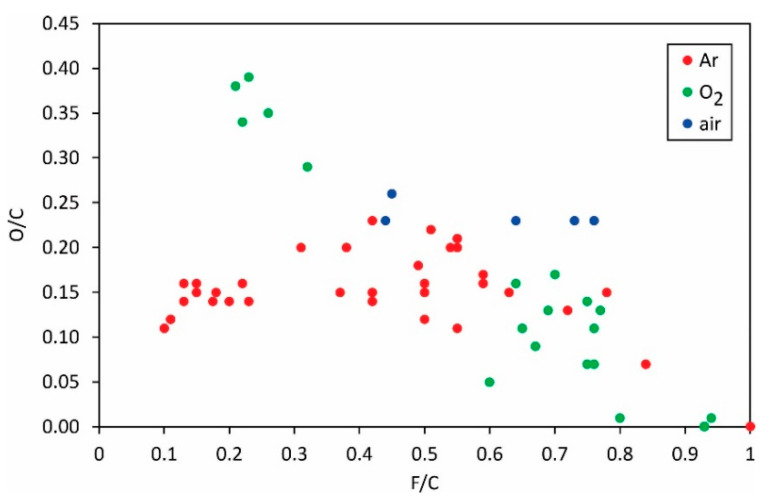
The O/C ratio versus the F/C ratio.

The correlation between fluorine depletion and oxygen enrichment on the PVDF surface at various plasma conditions is shown in Figure 4.

The general trend is obvious only for the experiments with oxygen plasma: increasing the fluorine concentration in the surface film as probed by XPS will result in a decrease in oxygen concentration. Obviously, fluorine from the surface film is at least partially substituted with oxygen upon treatment of PVDF with oxygen plasma. For the other two gases (i.e., argon and air), the results are scattered so much that no general trend is observed. The scattering is explained by different mechanisms involved in surface chemistry upon treatment of this polymer with plasmas sustained at different discharge configurations. Figure 4 indicates a slight variation of the oxygen concentration versus the F/C ratio: the O/C is close to 0.15, practically irrespective of the F/C ratio. The observation can be explained by different mechanisms of interaction between argon plasma and the PVDF ratio, as illustrated in Figure 1, Figure 2 and Figure 3.

**Figure 5:**
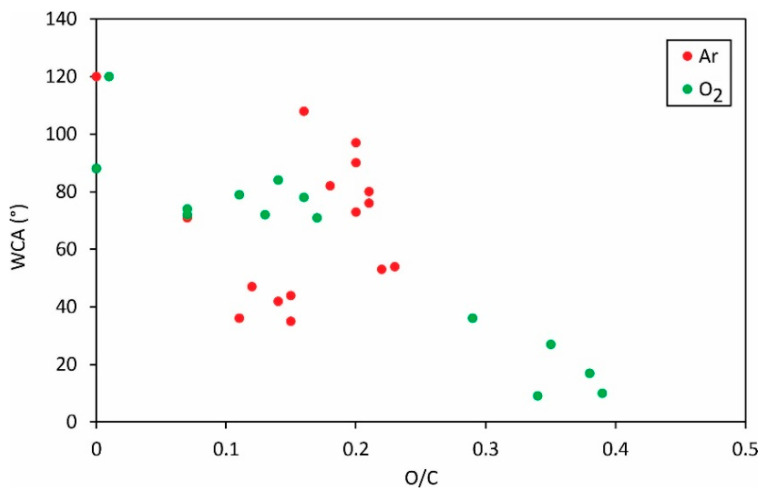
The static water contact angle versus the O/C ratio in the surface film as probed by XPS.

The correlation between the static water contact angle and the concentration of oxygen in the surface film probed by XPS is shown in Figure 5.

Again, the results are scattered, but the trend is obvious: a larger concentration of oxygen in the surface film results in a lower static water contact angle. The large oxygen concentration is therefore favorable for high surface wettability, the same as for most other polymers. The significant deviation of the measured points for argon plasma from the general trend is, again, explained by different mechanisms as illustrated in Figure 1, Figure 2 and Figure 3.

**Figure 6:**
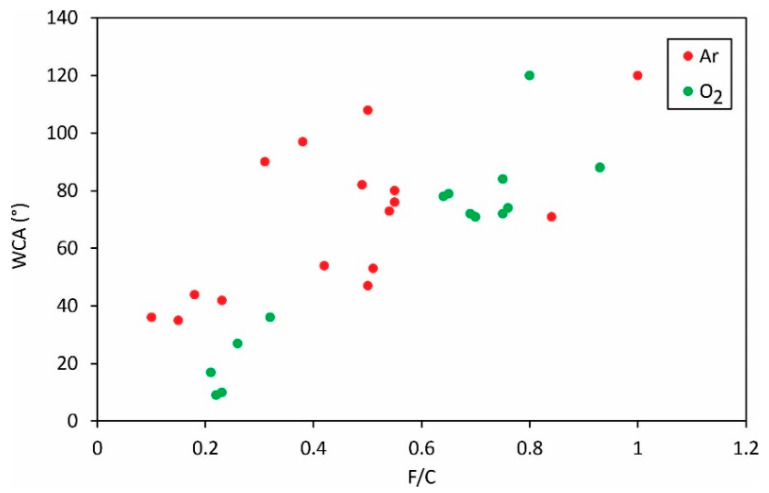
The static water contact angle versus the F/C ratio in the surface film as probed by XPS.

The static WCA versus the ratio between fluorine and carbon concentrations is plotted in Figure 6. Despite the large scattering of the reported results, the general observation is that the WCA increases with increasing F/C. Therefore, the defluorination is beneficial for increased wettability of this hydrophobic polymer.

**Figure 7:**
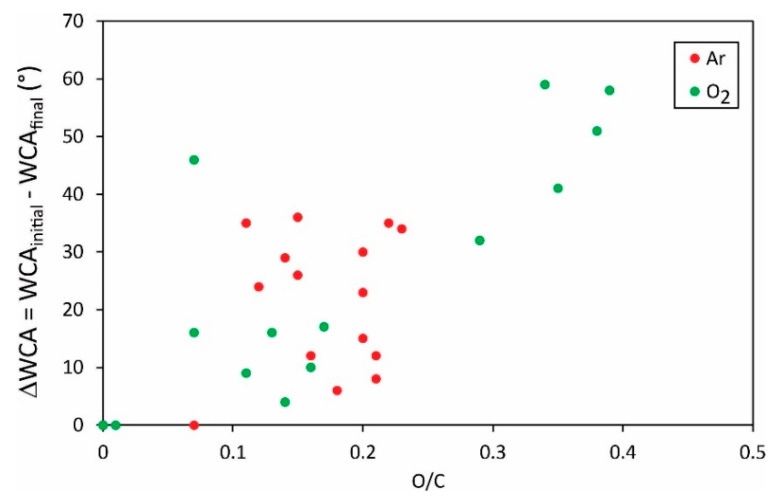
The change of the static water contact angle versus the O/C ratio in the surface film as probed by XPS.

**Figure 8:**
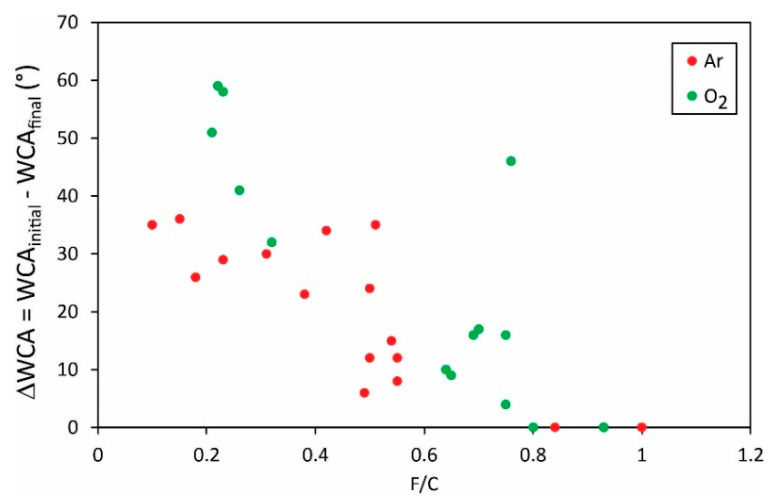
The change of the static water contact angle versus the O/C ratio in the surface film as probed by XPS.

Different authors reported various initial water contact angles, i.e., the static WCA for a sample before any plasma treatment. The WCAs after the plasma treatment as shown in Figure 5 and Figure 6 may, therefore, not be the most representative results. An important result may be the change in the water contact angle, i.e., the difference between the initial and the final water contact angle. The difference of the O/C versus the F/C ratio, respectively, is plotted in Figure 7 and Figure 8.

Vandencastelle et al. [35] reported the largest difference of almost 60°, but the majority of authors found the difference much smaller, about 40°. From this perspective, the hydrophilization of PVDF is not as effective as reported for many other polymers [36,37,38]. In fact, some authors reported the change in water contact angles close to 100° [39]. Plasma methods for optimal hydrophilization of PVDF are therefore yet to be discovered.

**Figure 9:**
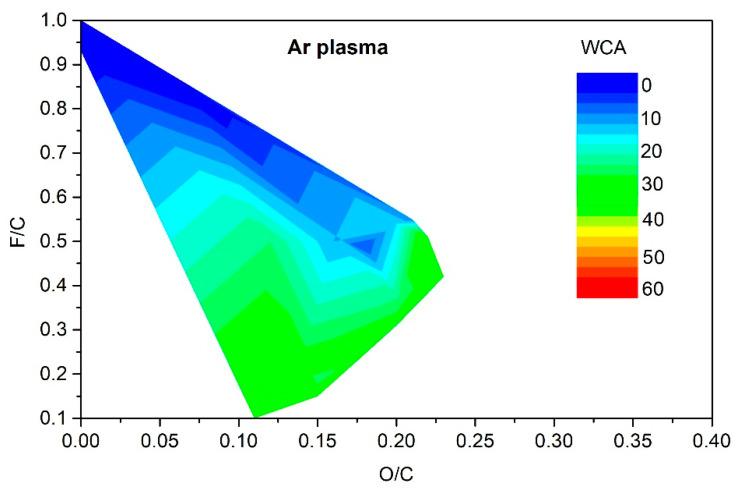
The change of the static water contact angle for various combinations of oxygen and fluorine surface concentrations obtained by argon-plasma treatments.

Figure 5, Figure 6, Figure 7 and Figure 8 reveal the variation of the static water contact angle versus the surface composition. The results are scattered because of various experimental conditions leading to a vague relationship between the oxygen and fluorine concentrations, as revealed in Figure 4. The range of achievable hydrophilization when using argon plasma is illustrated in Figure 9.

**Figure 10:**
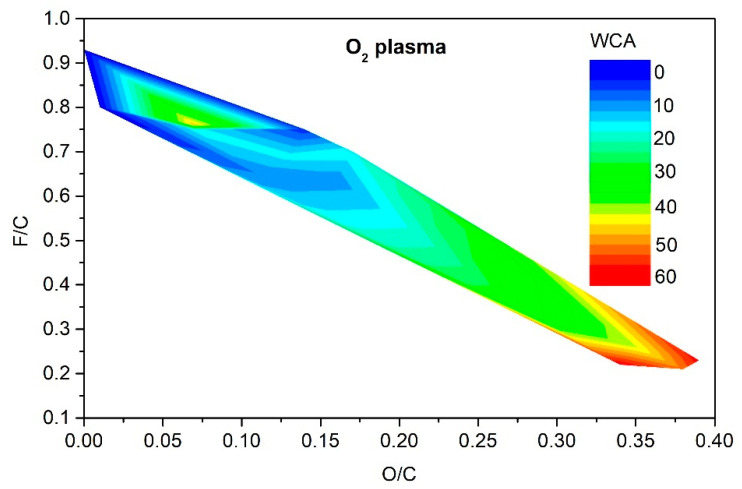
The change of the static water contact angle for various combinations of oxygen and fluorine surface concentrations obtained by oxygen plasma treatments.

As explained above, only moderate hydrophilization is achievable using argon plasma in the range of experimental conditions reported by different authors (Table 1). The range of O and F concentrations that enable such moderate hydrophilization is rather broad, as revealed in Figure 9. It is much narrower for the case of PVDF treatment with oxygen plasma. The resultant hydrophilization is illustrated in Figure 10. There is an islet of rather good hydrophilization at a rather large F/C and surprisingly low O/C ratio, whose origin is difficult to explain on the basis of available experimental details provided by the authors.

The surface finish of polymers treated with gaseous plasma depends on the fluxes and fluences of reactive gaseous species and radiation in the VUV range on the sample surface. Unfortunately, no author reported the fluxes. Instead, most reported the discharge power and the treatment time. The density of reactive plasma species depends on particularities of the experimental setup, and the dependence is not trivial, but generally, the density (and thus the flux) of the reactive species should increase with increasing power. The fluence is just a product of the flux and the treatment time, so a feasible parameter will be the product of the discharge power and the treatment time.

**Figure 11:**
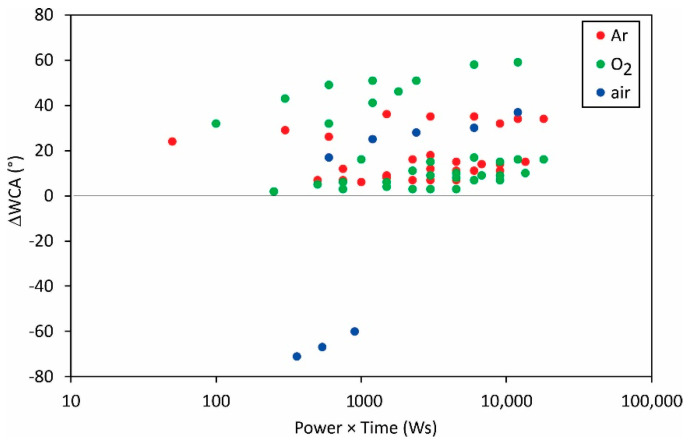
The difference between the initial static water contact angle and the WCA after the treatment versus the product of the reported discharge power and treatment time.

Figure 11 shows the change in the static water contact angle reported by various authors versus the product of the discharge power and the treatment time. No correlation could be drawn from the measured values as shown in Figure 11, so one may conclude that other parameters (peculiarities of the experimental setups) play a dominant role in surface wettability.

**Figure 12:**
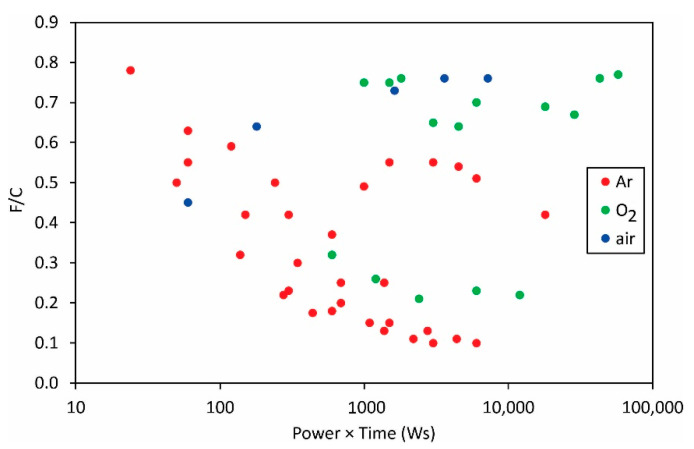
The F/C ratio versus the product of the reported discharge power and treatment time.

**Figure 13:**
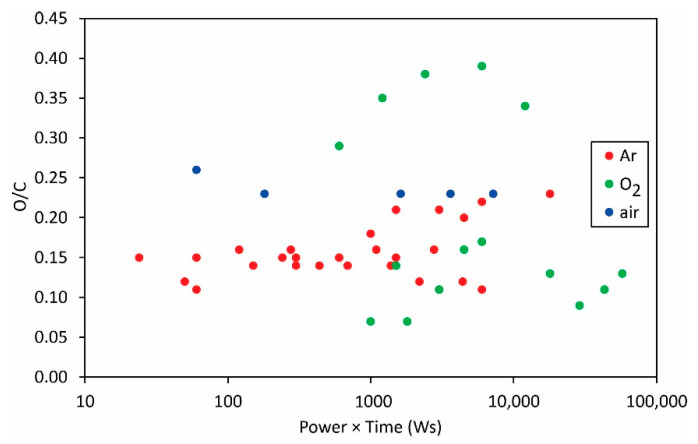
The O/C ratio versus the product of the reported discharge power and treatment time.

The same applies to the variation of the F/C and O/C ratios as deduced from XPS measurements (Figure 12 and Figure 13).

The measured points are so much scattered in Figure 12 and Figure 13 that it is obvious that the science of PVDF functionalization is still in its infancy. The scientific challenge will be the determination of the surface composition and wettability versus the fluences of the reactive species onto the polymer surface.

Finally, it is worth mentioning that most authors used either inert (argon) or reactive (usually oxygen) gas for plasma treatment of polymers. Despite the high purity of the gases, gaseous impurities are unavoidable in practical cases so argon plasma often comprises reactive species which are formed due to the efficient dissociation of impurity molecules in argon plasma. The density of impurity gases is low in a properly designed plasma reactor, but one can increase it by intentional introduction of a reactive gas into the plasma reactor filled with a noble gas. The introduction of small quantities of reactive gas in a controlled manner will enable additional flexibility of the processing parameters, especially as compared to treatments in oxygen or air, which may be too aggressive to obtain the desired surface finish.

## 6. Conclusions and Roadmap

The description of the mechanisms involved during surface activation of polyvinylidene fluoride by gaseous plasma treatment remains a scientific challenge. While the general concept is known, the peculiarities typical of this type of fluorinated polymer are still unclear. The defluorination, dehydrogenation and functionalization of the surface film with polar functional groups often occur simultaneously, especially when oxygen-containing plasma is used for polymer treatment. The amount of oxygen in the processing gas is difficult to control except when ultra-high vacuum compatible systems are used. The oxygen chemistry on the surface of PVDF depends on the degree of defluorination and dehydrogenation. As early as 1997, it was clearly demonstrated that the excessive dehydrogenization caused by treatment with energetic argon ions and VUV radiation does not allow for appreciating functionalization with polar functional groups and thus appropriate hydrophilicity of this polymer. Water contact angles below 15° were reported only by one group that used oxygen plasma treatment at low power density. Other groups reported rather insufficient improvement of the surface wettability by oxygen–plasma treatment that is explained by excessive etching of the modified surface film. The key scientific challenge is studying the surface kinetics using small fluxes of reactive species. The theoretical predictions about the substitution of hydrogen atoms from the polymer surface with hydroxyl groups are yet to be proven experimentally for this type of polymer. Polyvinylidene fluoride is a material of choice in niche applications, so there is a need to develop techniques for optimal surface activation of high reliability, preferably within a rather broad range of processing parameters. Such a reliable activation that would lead to an almost super-hydrophilic surface finish is yet to be discovered.

## Figures and Tables

**Table 1:** Summary of results reported by various authors who have probed argon-plasma treatment.

Author	Ref	Pressure (Pa)	Discharge	Frequency (MHz)	Power (W)	Time (s)	WCA Before (°)	WCA After (°)	WCA Change (°)	F/C	O/C	Material Type
Yalcin-kaya	[13]	low	MW	2450		300	120	0	0			electrospun
Duca	[6]	13.3	RF	13.56		0	71	71	0	0.84	0.07	foil
Duca	[6]	13.3	RF	13.56	100	0.5	71	47	24	0.5	0.12	foil
Duca	[6]	13.3	RF	13.56	100	3	71	42	29	0.23	0.14	foil
Duca	[6]	13.3	RF	13.56	100	6	71	44	26	0.18	0.15	foil
Duca	[6]	13.3	RF	13.56	100	15	71	35	36	0.15	0.15	foil
Duca	[6]	13.3	RF	13.56	100	30	71	36	35	0.1	0.11	foil
Duca	[6]	13.3	RF	13.56	100	60	71	36	35	0.1	0.11	foil
Duca	[6]	13.3	RF	13.56	73	0				0.84	0.07	foil
Duca	[6]	13.3	RF	13.56	73	6				0.175	0.14	foil
Duca	[6]	13.3	RF	13.56	73	15				0.15	0.16	foil
Duca	[6]	13.3	RF	13.56	73	30				0.11	0.12	foil
Duca	[6]	13.3	RF	13.56	73	60				0.11	0.12	foil
Duca	[6]	13.3	RF	13.56	46	0				0.84	0.07	foil
Duca	[6]	13.3	RF	13.56	46	6				0.22	0.16	foil
Duca	[6]	13.3	RF	13.56	46	15				0.20	0.14	foil
Duca	[6]	13.3	RF	13.56	46	30				0.13	0.14	foil
Duca	[6]	13.3	RF	13.56	46	60				0.13	0.16	foil
Duca	[6]	13.3	RF	13.56	23	0				0.84	0.07	foil
Duca	[6]	13.3	RF	13.56	23	6				0.32		foil
Duca	[6]	13.3	RF	13.56	23	15				0.30		foil
Duca	[6]	13.3	RF	13.56	23	30				0.25		foil
Duca	[6]	13.3	RF	13.56	23	60				0.25		foil
Duca	[6]	13.3	RF	13.56	10	0				0.84	0.07	foil
Duca	[6]	13.3	RF	13.56	10	6				0.55	0.11	foil
Duca	[6]	13.3	RF	13.56	10	15				0.42	0.14	foil
Duca	[6]	13.3	RF	13.56	10	30				0.42	0.15	foil
Duca	[6]	13.3	RF	13.56	10	60				0.37	0.15	foil
Duca	[6]	13.3	RF	13.56	4	0				0.84	0.07	foil
Duca	[6]	13.3	RF	13.56	4	6				0.78	0.15	foil
Duca	[6]	13.3	RF	13.56	4	15				0.63	0.15	foil
Duca	[6]	13.3	RF	13.56	4	30				0.59	0.16	foil
Duca	[6]	13.3	RF	13.56	4	60				0.5	0.15	foil
Park	[10]	13.3	RF	13.56		0	88	88	0	0.93	0	foil
Park	[10]	13.3	RF	13.56	100	10	88	82	6	0.49	0.18	foil
Park	[10]	13.3	RF	13.56	100	30	88	70	18			foil
Park	[10]	13.3	RF	13.56	100	60	88	53	35	0.51	0.22	foil
Park	[10]	13.3	RF	13.56	100	90	88	56	32			foil
Park	[10]	13.3	RF	13.56	100	120	88	54	34			foil
Park	[10]	13.3	RF	13.56	100	180	88	54	34	0.42	0.23	foil
Park	[10]	13.3	RF	13.56		0	88	88	0	0.93	0	foil
Park	[10]	13.3	RF	13.56	75	10	88	76	12			foil
Park	[10]	13.3	RF	13.56	75	30	88	72	16			foil
Park	[10]	13.3	RF	13.56	75	60	88	73	15	0.54	0.20	foil
Park	[10]	13.3	RF	13.56	75	90	88	74	14			foil
Park	[10]	13.3	RF	13.56	75	120	88	74	14			foil
Park	[10]	13.3	RF	13.56	75	180	88	73	15			foil
Park	[10]	13.3	RF	13.56		0	88	88	0	0.93	0	foil
Park	[10]	13.3	RF	13.56	50	10	88	81	7			foil
Park	[10]	13.3	RF	13.56	50	30	88	79	9			foil
Park	[10]	13.3	RF	13.56	50	60	88	76	12	0.55	0.21	foil
Park	[10]	13.3	RF	13.56	50	90	88	77	11			foil
Park	[10]	13.3	RF	13.56	50	120	88	77	11			foil
Park	[10]	13.3	RF	13.56	50	180	88	77	11			foil
Park	[10]	13.3	RF	13.56		0	88	88	0	0.93	0	foil
Park	[10]	13.3	RF	13.56	25	10	88	86	2			foil
Park	[10]	13.3	RF	13.56	25	30	88	81	7			foil
Park	[10]	13.3	RF	13.56	25	60	88	80	8	0.55	0.21	foil
Park	[10]	13.3	RF	13.56	25	90	88	81	7			foil
Park	[10]	13.3	RF	13.56	25	120	88	81	7			foil
Park	[10]	13.3	RF	13.56	25	180	88	81	7			foil
Akashi	[14]	10^5^	HF	0.02		0	120	120	0	1	0	membrane
Akashi	[14]	10^5^	HF	0.02	4 kV	30	120	114	6			membrane
Akashi	[14]	10^5^	HF	0.02	4 kV	60	120	108	12	0.50	0.16	membrane
Akashi	[14]	10^5^	HF	0.02	4 kV	120	120	102	18			membrane
Akashi	[14]	10^5^	HF	0.02	4 kV	180	120	97	23	0.38	0.20	membrane
Akashi	[14]	10^5^	HF	0.02	4 kV	240	120	93	27			membrane
Akashi	[14]	10^5^	HF	0.02	4 kV	300	120	90	30	0.31	0.20	membrane
Akashi	[15]	10^5^	HF	0.02		0				0.72	0.13	membrane
Akashi	[15]	10^5^	HF	0.02	4 kV	60				0.59	0.16	membrane
Akashi	[15]	10^5^	HF	0.02	4 kV	180				0.59	0.17	membrane
Akashi	[15]	10^5^	HF	0.02	4 kV	300				0.55	0.20	membrane

**Table 2:** Summary of results reported by various authors who have probed oxygen-plasma treatment.

Author	Ref	Pressure (Pa)	Discharge	Frequency (MHz)	Power (W)	Time (s)	WCA Before (°)	WCA After (°)	WCA Change (°)	F/C	O/C	Material Type
Vandencastelle	[20]	7	RF	13.56		0	68			0.6	0.05	foil
Vandencastelle	[20]	7	RF	13.56	20	30	68	36	32	0.32	0.29	foil
Vandencastelle	[20]	7	RF	13.56	20	60	68	27	41	0.26	0.35	foil
Vandencastelle	[20]	7	RF	13.56	20	120	68	17	51	0.21	0.38	foil
Vandencastelle	[20]	7	RF	13.56	20	300	68	10	58	0.23	0.39	foil
Vandencastelle	[20]	7	RF	13.56	20	600	68	9	59	0.22	0.34	foil
Park	[10]	13.3	RF	13.56		0	88	88	0	0.93	0	foil
Park	[10]	13.3	RF	13.56	100	10	88	72	16	0.75	0.07	foil
Park	[10]	13.3	RF	13.56	100	30	88	73	15			foil
Park	[10]	13.3	RF	13.56	100	60	88	71	17	0.70	0.17	foil
Park	[10]	13.3	RF	13.56	100	90	88	73	15			foil
Park	[10]	13.3	RF	13.56	100	120	88	72	16			foil
Park	[10]	13.3	RF	13.56	100	180	88	72	16	0.69	0.13	foil
Park	[10]	13.3	RF	13.56		0	88	88	0	0.93	0	foil
Park	[10]	13.3	RF	13.56	75	10	88	82	6			foil
Park	[10]	13.3	RF	13.56	75	30	88	77	11			foil
Park	[10]	13.3	RF	13.56	75	60	88	78	10	0.64	0.16	foil
Park	[10]	13.3	RF	13.56	75	90	88	79	9			foil
Park	[10]	13.3	RF	13.56	75	120	88	79	9			foil
Park	[10]	13.3	RF	13.56	75	180	88	78	10			foil
Park	[10]	13.3	RF	13.56		0	88	88	0	0.93	0	foil
Park	[10]	13.3	RF	13.56	50	10	88	83	5			foil
Park	[10]	13.3	RF	13.56	50	30	88	82	6			foil
Park	[10]	13.3	RF	13.56	50	60	88	79	9	0.65	0.11	foil
Park	[10]	13.3	RF	13.56	50	90	88	80	8			foil
Park	[10]	13.3	RF	13.56	50	120	88	81	7			foil
Park	[10]	13.3	RF	13.56	50	180	88	81	7			foil
Park	[10]	13.3	RF	13.56		0	88	88	0	0.93	0	foil
Park	[10]	13.3	RF	13.56	25	10	88	86	2			foil
Park	[10]	13.3	RF	13.56	25	30	88	85	3			foil
Park	[10]	13.3	RF	13.56	25	60	88	84	4	0.75	0.14	foil
Park	[10]	13.3	RF	13.56	25	90	88	85	3			foil
Park	[10]	13.3	RF	13.56	25	120	88	85	3			foil
Park	[10]	13.3	RF	13.56	25	180	88	85	3			foil
Kim	[21]	6.7	RF	13.56	10	0	120	120	0	0.8	0.01	membrane
Kim	[21]	6.7	RF	13.56	10	10	120	88	32			membrane
Kim	[21]	6.7	RF	13.56	10	30	120	77	43			membrane
Kim	[21]	6.7	RF	13.56	10	60	120	71	49			membrane
Kim	[21]	6.7	RF	13.56	10	120	120	69	51			membrane
Kim	[21]	6.7	RF	13.56	10	180	120	74	46	0.76	0.07	membrane
Correia	[23]	20	RF	13.56	0	120				0.94	0.01	electrospun
Correia	[23]	20	RF	13.56	240	120				0.67	0.09	electrospun
Correia	[23]	20	RF	13.56	360	120				0.76	0.11	electrospun
Correia	[23]	20	RF	13.56	480	120				0.77	0.13	electrospun
Jeong	[25]	2.7	RF	13.56	62	0				0.5–EDX	0–EDX	membrane
Jeong	[25]	2.7	RF	13.56	62	1800				1.0–EDX	0.1–EDX	membrane
Jeong	[25]	2.7	RF	13.56	62	7200				0.6–EDX	0.05–EDX	membrane

**Table 3:** Summary of results reported by various authors who have probed plasmas other than oxygen or argon.

Author	Ref	Gas	Pressure (Pa)	Discharge	Frequency (MHz)	Power (W)	Time (s)	WCA Before (°)	WCA After (°)	WCA Change (°)	F/C	O/C	Material Type
Gopakumar	[31]	CO_2_	13	MW	2450	1000	300	140	100	40			electrospun
Kormunda	[33]	air	10^5^	Plannar DBD	0.003	120	0	69	69	0	0.44	0.23	electrospun
Kormunda	[33]	air	10^5^	Plannar DBD	0.003	120	0.5				0.45	0.26	electrospun
Kormunda	[33]	air	10^5^	Plannar DBD	0.003	120	1.5				0.64	0.23	electrospun
Kormunda	[33]	air	10^5^	Plannar DBD	0.003	120	3	69	140	−71			electrospun
Kormunda	[33]	air	10^5^	Plannar DBD	0.003	120	4.5	69	136	−67			electrospun
Kormunda	[33]	air	10^5^	Plannar DBD	0.003	120	7.5	69	129	−60			electrospun
Kormunda	[33]	air	10^5^	Plannar DBD	0.003	120	13.5				0.73	0.23	electrospun
Kormunda	[33]	air	10^5^	Plannar DBD	0.003	120	30				0.76	0.23	electrospun
Kormunda	[33]	air	10^5^	Plannar DBD	0.003	120	60				0.76	0.23	electrospun
Vandencasteele	[20]	N_2_	7	RF	13.56	20	0	55	55	0			foil
Vandencasteele	[20]	N_2_	7	RF	13.56	20	30	55	38	17			foil
Vandencasteele	[20]	N_2_	7	RF	13.56	20	60	55	30	25			foil
Vandencasteele	[20]	N_2_	7	RF	13.56	20	120	55	27	28			foil
Vandencasteele	[20]	N_2_	7	RF	13.56	20	300	55	25	30			foil
Vandencasteele	[20]	N_2_	7	RF	13.56	20	600	55	18	37			foil

## Data Availability

Not applicable.
